# Microsatellite Instability in Sporadic Colorectal Malignancy: A Pilot Study from Northern India

**DOI:** 10.31557/APJCP.2021.22.7.2279

**Published:** 2021-07

**Authors:** Smita Chauha, Suneed Kumar, Pradyumn Singh, Nuzhat Husain, Shakeel Masood

**Affiliations:** 1 *Department of Surgical Gastroenterology, Dr. Ram Manohar Lohia Institute of Medical Sciences, Lucknow, India. *; 2 *Department of Pathology, Dr. Ram Manohar Lohia Institute of Medical Sciences, Lucknow, India. *

**Keywords:** Microsatellite instability, mismatch repair, colorectal cancer, PCR

## Abstract

**Background:**

Three molecular pathways are described as the genetic basis of colorectal tumorigenesis. Among these, microsatellite instability (MSI) has shown greatest promise in serving as a biomarker to determine disease aggression by tumour biology, recurrence, and response to chemotherapy.

**Methodology:**

This prospective observational pilot study included patients of colorectal cancers, in a population subset coming to a tertiary care hospital in northern India, who were operated with curative or palliative intent over a period of one year and followed up for a maximum of 55 months. The post-operative pathological assessment was done for MSI status using PCR technique, and an attempt was made to evaluate its correlation with conventional clinical and histological parameters, early recurrences, disease-free survival and overall survival in comparison to MSS type tumours in sporadic cases of colorectal malignancies.

**Results:**

Out of 38 patients of colorectal cancer, 26 were included in the study. Male to female ratio was 7:6 (n=14:12). Mean age of presentation was 48±14.2 years. Incidence of MSI was n=4 (15.4%). On subgroup analysis, age of presentation (p=0.044) and evidence of perineural invasion (p=0.017) was found to have significant statistical association with MSI tumour biology. Recurrence was seen in seven of the seventeen patients who previously had no synchronous or metastatic disease (41.2%). The mean disease-free survival for MSS was 21.32 months and was 25.25 months for MSI group which was statistically insignificant (p = 0.277). Out of four MSI tumour biology patients one was alive and without recurrence at 47 months. While the other two were alive and without recurrence till 27 months of follow-up.

**Conclusion:**

Age and perineural invasion showed statistically significant association with MSI tumour biology. Due to the small sample size statistical significance was not established with site, recurrence rate, DFS and OS.

## Introduction

Three molecular pathways are described as the genetic basis of colorectal tumorigenesis. Among these, microsatellite instability (MSI) has shown greatest promise in serving as a biomarker to determine disease aggression by tumour biology, recurrence, and response to chemotherapy. 

Colorectal cancer (CRC) is the third most common cancer in men, second most common cancer in women (GLOBOCAN, 2008)and the second leading cause of cancer death in the world. In India, the annual incidence for colon cancer in men is 4.4 per 100,000, and 3.9 per 100,000 for women (NRCP, 2013). CRC is a heterogeneous disease, and at the molecular level previous research has shown three major pathways in colorectal carcinogenesis (van Engeland et al., 2011); namely CpG island methylator phenotype (CIMP), chromosomal instability (CIN) and Microsatellite instability (MSI) pathway. Among these, DNA methylation is a particularly interesting event since it occurs early in carcinogenesis. Various methylated genes engaged in each one of the pathways may be evaluated to serve as biomarkers. 

Microsatellites (MS) are simple repetitive DNA sequences scattered throughout the genome, composed of 1 to 6 base pair units that may repeat up to 100 times. They are inherently hyper mutable because of their propensity for strand slippage during DNA replication. The correction of the resulting insertion/deletion loops requires the intact function of the DNA mismatch repair (MMR) system. With loss of MMR function, insertion/deletion loops are not repaired, resulting in the variable expansion or contraction of microsatellites. This phenomenon is referred to as Microsatellite instability (MSI) (Fujiwara et al., 1998; Horvat et al., 2011). MSI is detected in about 15% of all colorectal cancers; 3% of these are associated with Lynch syndrome and the other 12% are sporadic (Lynch et al., 2002; Popat et al., 2005).

This prospective observational pilot study was undertaken to determine the incidence of MSI positivity, its correlation with conventional clinical and histological parameters, early recurrence, disease free survival and overall survival as compared to microsatellite stable tumours, in cases of sporadic colorectal cancers, in a population subset coming to a tertiary care hospital in northern India.

## Materials and Methods

*Study design*

During a time-bound study period of one year (March 2016 – March 2017), 26 patients with pathologically confirmed colorectal carcinoma, presenting at different stages of the disease, underwent surgical intervention with curative or palliative intent. With due informed and written consent, they were included in the study design. The Institutional Research Committee approved the study as a pilot intramural project. 

The study was designed as a prospective observational study, with cases recruited during the initial period (one year), and later followed up for maximum of 55 months thereafter. Demographic, clinical and tumour related information was noted for each patient, along with the indication and intent for surgery. The tumours were classified according to TNM staging as per AJCC 8th edition. Curative intent surgeries included a standard D2 level lymphadenectomy; while in the palliative setting, this was avoided. 

The resected specimen underwent histological typing on the Hematoxylin & Eosin stained slides. Formalin-fixed paraffin embedded (PPFE) or fresh tissue samples were collected from tumour site and adjacent normal colonic epithelium, and used to make two blocks for each patient. 

*DNA Isolation from FFPE Tissue*

DNA from paraffin embedded sections was isolated by FFPE tissue DNA kit (Qiagen, USA). Briefly, 5-6 sections of 8μm thickness were cut and dissolved in 1ml xylene and the pellet washed with 1ml ethanol. After evaporation of excess ethanol, pellet was resuspended in 180μl Buffer ATL and 20μl proteinase K followed by incubation at 56^o^C for 1 hour and at 90^o^C for 1 hour. After incubation 200μl of 100% ethanol was added. Suspension was transferred to column tube and centrifuged followed by washing with wash buffer 1 and 2. Finally DNA was eluted in 60μl of elution buffer.

Tissue DNA concentration measurement: Concentration of the isolated DNA samples was measured by taking the absorbance at 260nm on Nanodrop (DeNovix, DS-11 Spectrophotometer).

*Multiplex PCR for Microsatellite Analysis*

The MSI analysis were carried out using multiplex PCR with fluorescently labelled primers, included in the MSI Analysis System kit, Version 1.2 (Cat no# MD1641, Promega Corp. Madison, WI, USA) for amplification of five mononucleotide repeat markers (NR-21, BAT-26, BAT-25, NR-24, MONO-27) and two pentanucleotide repeat markers (Penta C and Penta D). The PCR reactions were performed in a 10μl volume containing 2ng DNA, 1× Gold ST*R buffer (Promega), 1× MSI Analysis System Primer Pair Mix (Promega), 0.15μl AmpliTaq Gold DNA Polymerase (5U/μl) (Applied Biosystem, USA) and nuclease free H2O (Qiagen, USA). The PCR was performed using a GeneAmp® System 9600 Thermal Cycler (Veriti Thermal Cycler, Applied Biosystem, USA) and the following PCR programme: 95^o^C for 11 min, 96^o^C for 1 min, 10 × (ramp 100% to 94^o^C 30s, ramp 29% to 58% 30s, ramp 23% To 70^o^C 1min) and 60^o^C for 30 min and final hold at 4^o^C. 

*Microsatellite Fragment Analysis*

A loading cocktail was prepared by mixing 9.5μl Hi-DiTM Formamide (Applied Biosystems, Foster City, CA, USA) with 0.5μl of an internal lane standard, ILS600 (Promega), for each sample. After vortexing the loading cocktail it was aliquoted into a 96-well plate. Subsequently, 1μl of each PCR product was added to each well with loading cocktail. The 96-well which was then shaken briefly to remove air bubbles followed by denaturation of the samples at 95^o^C for 3min and then snap chilled in the ice for 3 min. The 96-well plate was loaded for capillary electrophoresis in an ABI 3500 instrument (Applied Biosystems, USA) for fragment analysis according to the instructions from the manufacturer (Promega). 

Data was analysed using GeneMapper^®^ software Version 5 (Applied Biosystems, USA) Samples were labelled MSI-High if two or more markers showed instability (Figure 3) , MSI-Low if one marker showed instability and MSS (microsatellite stable) if no marker displayed instability (Figure 4), in accordance with the NCI guidelines.

*Adjuvant Therapy and Follow up*

The patients were referred for adjuvant chemotherapy (FOLFOX regimen) after postoperative recovery. Select patients also received adjuvant radiotherapy as needed. In certain patients adjuvant chemotherapy was deferred (patient refused or low risk patients) and they were kept on close surveillance.

Follow-up protocol included, 3 monthly serum Carcino-embryonic antigen (CEA) level estimation, annual abdominal computerised tomography (CT) and biannual colonoscopy. (National Comprehensive Cancer Network -NCCN guidelines).

*Statistical Analysis*

Data analysis was done using SPSS Software for Windows v 22.0 (IBM, Armonk, NY) Descriptive statistics for numerical data was by median or mean ± standard deviation. Categorical data were described as percentages. Univariate analysis was performed by chi-squared test, keeping a two-tailed p-value <0.05 as significant. Acceptable type II error was 80%. 

## Results

Thirty-eight patients underwent resection for colorectal cancer in the department during the study duration, of which 26 consented and were included in the study. The male to female ratio was 7:6. Mean age of presentation was 48 ± 14.19 years and 50% of study population was ≥ 50 years of age. The overall demographics, histopathology and tumour characteristics are summarised in Table 1.

*Microsatellite Status*

Four of the study cohort of 26 patients tested positive for microsatellite instability (15.4%) with MSI-H phenotype in 3 (11.5%) and MSI-L in one (3.8%). All other patients were confirmed as MSS type. Among the MSI-H group, there were two female and one male patient; with ages ranging from 29 to 50 years. All these three patients had bulky right sided proximal colonic tumours, that required right hemicolectomy. The single patient with MSI-L phenotype was a 45 year-old male presenting with a locally advanced lower one-third rectal tumour requiring abdomino-perineal resection. 

*Adjuvant Therapy*

Seventeen patients (65.38%) received Capecitabine and Oxaliplatin based adjuvant chemotherapy (FOLFOX). Nine patients did not receive adjuvant chemotherapy. Among these, four patients succumbed prior to initiation of chemotherapy due to advanced disease or early disease progression. (Three patients were stage IV, one was stage III). Four patients in stage II were not administered chemotherapy; two since they were in the low-risk group and the other two patients (MSI-H phenotypes) refused chemotherapy due to personal reasons. One was Stage 1 patient post NACT, who had an early recurrence and death.

*Disease recurrence was computed from the patients *who underwent curative resection for cancer (n=17). Recurrence was found in seven patients (41.17%); all being distant instead of locoregional. These patients were given palliative chemo-radiation as deemed suitable. Seven patients were alive at the last follow up of 55 months. Of these one was Stage-3 MSI-H type patient who was alive without recurrence. The other two MSI-H type Stage-2 patients were alive and without recurrence till 27 months of index surgery after which they were lost to follow-up.

**Figure 1:**
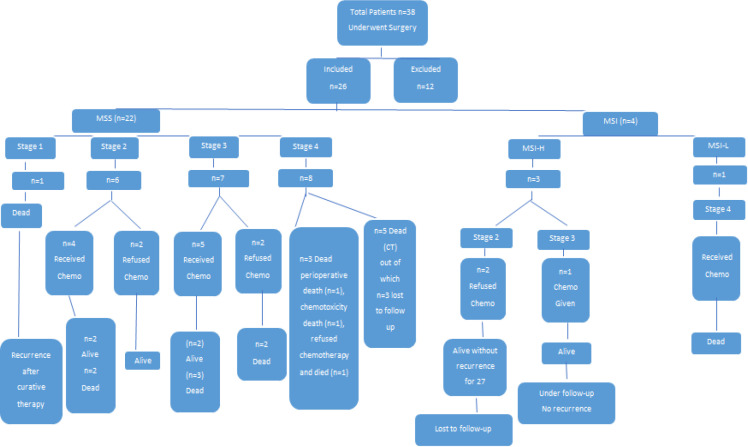
Stage Wise Outcome and Follow-up of MSS Patients

*Stage wise outcome and follow-up of MSS patients (* Table-1 *) and (* Figure -1 *)*

Of 22 MSS patients the lone patient in Stage I was operated electively and received adjuvant chemotherapy. However, patient had early recurrence and succumbed to the disease in 10 months.

There were 6 patients of stage II who underwent elective surgery with curative intent of which 4 received adjuvant chemotherapy while 2 refused the same. During follow up two of the adjuvant chemotherapy group patients had recurrence and succumbed to their illness at 29 month and 40 months. The other 4 were alive and without recurrence till last follow up.

There were 7 patients of Stage III in the MSS group of whom 6 had elective and 1 underwent emergency surgery. There was one perioperative mortality. 5 of the remaining 6 received adjuvant chemotherapy. 2 in the adjuvant chemotherapy group were still alive at last follow up and the other 4 patients had recurrences and succumbed to their illnesses at 16, 30, 43 and 51 months respectively. 

Of the 8 cases with Stage IV disease in the MSS group only 2 had been operated electively and peritoneal metastasis detected during surgery, while the other 6 had required emergency surgery for complications like obstruction, perforation or bleed. Of these 8 patients 3 were lost to follow up within 6 months of initiation of chemotherapy. Of the remaining 5 – 1 was a perioperative death due to mesenteric vascular thrombosis, the other died within 45 days of surgery due to chemotoxicity and another in 14 months following surgery, this patient had refused chemotherapy due to personal reasons. The 2 Stage IV cases who received adjuvant chemotherapy also succumbed to their illness in 15 & 30 months.

*Stage wise outcome & follow up of MSI patients (* Table 1 *) and (* Figure 1 *)*

There were 4 patients with MSI tumour biology of whom 3 were MSI-high and one MSI-Low type. 2 of the MSI-H type patients had stage-II disease while one had stage-III disease. All the 3 had been operated electively with curative intent. Both the Stage-II patients refused chemotherapy and were alive without recurrence till 27 months post index surgery after which they were lost to follow-up. The lone patient of Stage-III received chemotherapy and is alive with no recurrence at 47 months follow-up. Single MSI-Low type patient had Stage-IV disease. This patient received chemotherapy and succumbed to his illness after 28 month of follow up.

In the cumulative follow up of both groups three patients were lost to follow up after the initial six months post chemotherapy. There were two mortalities within 30 days of surgery: one due to a cardiac event and the other related to short bowel syndrome secondary to superior mesenteric vascular thrombosis. Another patient died during chemotherapy on the forty-fifth day post-surgery. The remaining 20 patients were followed up for a mean duration of 35.1 months and a maximum of 55 months from surgery. Eleven patients (55%) out of 20 had succumbed to illness during surveillance, 2-MSI-H type stage -2 patients were lost to follow up after 27 months. While 7 (35%) patients including 1-MSI-H type stage 3 patient were still alive and without recurrence at the time of last follow up. 

The mean disease-free survival for MSS was 21.32 months and was 25.25 months for MSI group which was statistically insignificant (p = 0.277) Overall survival was statistically insignificant for MSI group which was 32.25 months as compared to MSS group which was about 26.11 months (p = 0.277). 

*Univariate and Sub-group Analysis*

On subgroup analysis, age of presentation and evidence of perineural invasion was found to be statistically significant variables correlating to MSI status. The details of various parameters are shown in Table 2. All the cases (n=4) with MSI (high; n=3 / low; n=1) status were 50 years or less in age at presentation. There was no associated positive family history among the MSI cases, thereby ruling out possibility of Lynch syndrome. Most of the tumours with microsatellite instability presented as bulky right colonic malignancies.

Univariate analysis with MMR status as the dependant variable showed no statistically significant parameter; hence multiple logistic regression was not applied. The Odds ratio (OR) of tumour site was found to be higher than other variables, indicating the correlation of right sided colonic malignancies with MSI status, albeit statistically insignificant.

The MSI-L patient who presented with stage IV disease succumbed during surveillance; remaining 3 MSI-H phenotypes were alive and well without any metachronous lesion till 27 months from index surgery thereafter 2 stage-2 patients lost to follow-up while the lone stage-3 patient was alive and free of recurrence till last follow-up (47 months). Chemotherapy was administered to the stage III (MSI-H) and stage IV (MSI-L) lesions. Despite no adjuvant therapy in the two MSI-H stage II tumours, there was no evidence of recurrence till 27 months of follow-up (Table 3).

## Discussion

Three molecular pathways are described as genetic basis of colorectal tumorigenesis. The chromosomal instability (CIN) pathway characterised by the *Adenomatous Polyposis Coli (APC)* gene mutation was one of the earliest described models (Fearon and Vogelstein, 1990), and ascribes to both inherited and sporadic cancers. The hypermethylation phenotype or CpG island hypermethylation phenotype (CIMP) pathway was the latest to be described, and is characterised by the development of serrated hyperplastic colonic polyps (van Engeland et al., 2011; Weisenberger et al., 2006). The mismatch repair (MMR) pathway was initially described in colorectal cancers arising in relation to Lynch syndrome. It is typified by the dysfunction of mismatch repair genes and accumulation of microsatellites (Boland et al., 1998; Lynch et al., 2002).

Five genes that comprise the MMR pathway are MLH1 (MutL homolog 1; chromosome 3p22.2), MSH2 (MutS homolog 2; chromosome 2p21.16), MSH6 (MutS homolog 6; chromosome 2p16.3), PMS2 (post meiotic segregation 2; chromosome 7p22.1) and the PMS1 gene. They function as heterodimeric protein complexes to maintain genomic integrity (Moreira et al., 2012). The errors generated during DNA replication such as base-substitution and insertion-deletion mismatches are corrected by the complex. Inactivation of both alleles of any genes leads to defective MMR function; this can be accomplished by germline mutations (as in Lynch syndrome), somatic mutations or epigenetic alterations (Koessler et al., 2008). Consequent to these changes there is accumulation of repeated, abnormal, short sequences of nucleotide bases, called microsatellites (Shibata et al., 1994; Fujiwara et al., 1998). Tumours with such genetic alterations are said to have microsatellite instability.

**Figure 2:**
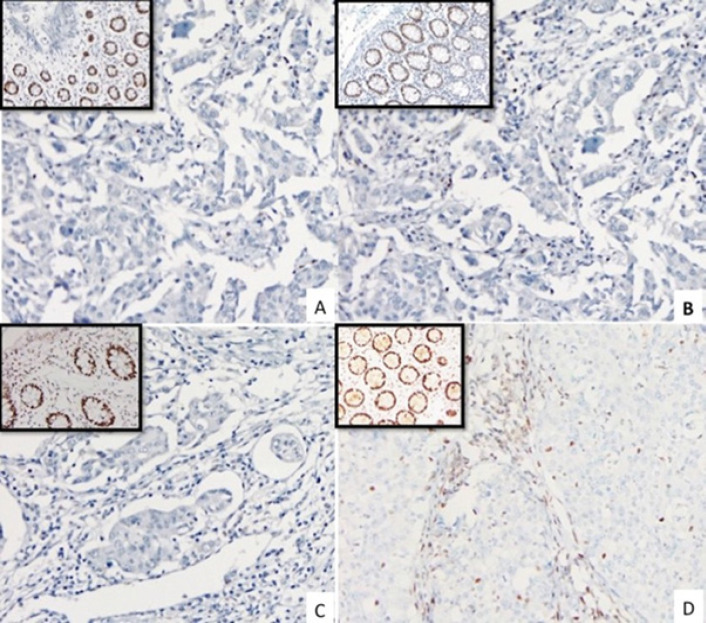
Microscopic pictures of Immunohistochemistry Analysis. (200X). A, Loss of MLH 1; B, Loss of PMS 2; C, Loss of MSH 2; D, Loss of MSH 6. Respective controls shown in insets

Testing for MSI status by polymerase chain reaction (PCR) is done to amplify a standard panel of DNA sequences containing nucleotide repeats. Three dinucleotide repeats and two mononucleotide repeats are the standard test panel for MSI (Hatch et al., 2005; Niu et al., 2014). A tumour is labelled MSI-H when at least two (40 percent) nucleotide repeats are affected by instability. Other tumours showing instability in <40 percent of the markers are referred to as MSI-low (MSI-L). If none of the markers show any instability the tumour is said to be MSI-stable (MSS) (Boland et al., 1998). While almost all MSI-H tumours are MMR deficient, most MSI-L tumors have no detectable MMR defect (de la Chapelle et al., 2010). Immunohistochemistry (IHC) has also been used to identify the phenotype, with detection of altered proteins produced by the defective MMR genes (Figure 2). The mutations typically result in a truncated or lost MMR protein, which is shown as loss of staining on IHC (Shia et al., 2008). 

Sporadic tumors with MSI-H phenotype have characteristic clinic-pathologic features (Bonadona et al., 2011). They tend to occur in the proximal colon, are more often poorly differentiated, have a greater mucinous component and contain lymphocytic infiltration. Majority of tumors with MSI phenotype in our study presented as large bulky right sided tumors, similar to findings described in literature. 

MSI tumors are proven to have better long-term prognosis in comparison to MSS malignancies. The most prominent effect of improved outcomes with MSI status are evident in stage II and III tumours (Wright et al., 2005; Dubey et al., 2016; Mojarad et al., 2016); although there are few reports that extend the effect to other stages as well (Horvat et al., 2011; Copija et al., 2017; Gupta et al., 2018). These tumours have decreased sensitivity to the conventional first-line chemotherapeutic agents, like 5 Fluoro-Uracil (5 FU) (Arnold et al., 2003; Fischer et al., 2007). Additionally, MSI tumours, especially the MSI-H phenotype are found to have better prognosis, despite not being administered adjuvant chemotherapy (Hemminki et al., 2000; Ribick et al., 2003). In our study, with the three MSI-H patients presenting as Stage II or III; and both the stage II patients not receiving adjuvant therapy, the overall survival (OS) was statistically significant when compared with MSS tumour phenotype. 

MSI testing has been incorporated into the investigative protocol of Stage II CRC according to the National Comprehensive Cancer Network (NCCN) guidelines (Colon Cancer, Version 1.2019). This was based on a single large study showing the lack of benefit of adjuvant 5 FU based chemotherapy on MSI Stage II cancers (Sargent et al., 2010). Subsequent meta-analyses done among Stage II and III MSI versus MSS patients observed no statistically significant difference in overall outcomes, irrespective of chemotherapy status in MSI-H (des Guetz et al., 2009). A minor benefit of chemotherapy was suggested in MSI-H group compared to the MSS. However, MSI status was concluded to be predictive of non-response to 5 FU based chemotherapy in Stage II and III cancers (Jover et al., 2006; Guastadisegni et al., 2010; Webber et al., 2015).

The differences between MSI-H and MSI-L tumour biology were studied by many; with the broad conclusion that MSI-L had behaviour and long-term outcomes similar to MSS, and so were found to be significantly different from MSI-H tumours in terms of DFS and OS (Thibodeau et al., 1993; Popat et al., 2005; Koessler et al., 2008; Kim et al., 2016). Similar findings were noted in our study as well, wherein the one patient with MSI-L tumour biology presented as Stage IV disease with dissemination, and hence had a poor outcome compared to the three MSI-H patients. Many research groups have not made this distinction, and reported the entire MSI group with better prognosis compared to MSS group (Buckowitz et al., 2005; Mohan et al., 2016). In our study, there was a trend towards prolonged DFS which probably failed to show statistical significance due to loss of follow-up of the two stage-2 patients after 27 months. 

A significant proportion of our study patients presented with disseminated disease and could not be offered curative therapy. This depicts delayed presentation and aggressive biology of tumours in our region. Decreased incidence of stage IV colorectal cancer due to reduced metastatic potential of MSI tumours has been described (Malesci et al., 2007; French et al., 2008).

Aggressive tumour biology is responsible for the 26.92% survival of study population at last follow-up. The only stage I tumour patient succumbed to early disease recurrence. This could be explained by the presence of occult metastasis at time of surgery; leading to early post-operative recurrence in the absence of adjuvant therapy.

Our study is limited by small sample size owing to the time-bound nature of the study design. The patients were followed up for maximum of Fifty-Five months with significant number being lost to follow-up which is the weakness of study. However, as an interim analysis, this study reiterates the role of MSI testing in colorectal malignancy, especially in stage II and III tumours. MSI tumours may not be candidates for conventional adjuvant chemotherapy. 

**Table 1:** TNM and Stage Grouping

Parameters	Number (n)	Percentage (%)
T Stage		
T1	1	3.8
T2	2	7.7
T3	10	38.5
T4	13	50
N Stage		
N0	11	42.3
N1	5	19.2
N2	10	38.5
M Stage		
M0	19	73.1
M1	7	26.9
Stage Grouping		
Stage I	1	3.8
Stage II	8	30.8
Stage III	8	30.8
Stage IV	9	34.6

**Table 2:** Subgroup and Univariate Analysis

Parameters		MSI status		p-value	OR	95% CI
		MSS n (%)	MSI n (%)			
Sex	Male	12 (54.5)	2 (50)	0.867	1.2	0.142 – 10.119
	Female	10 (45.5)	2 (50)			
Age	≤50 years	10 (45.5)	4 (100)	0.044*	-	-
	>50 years	12 (54.5)	0 (0)			
Family History	Yes	1 (4.5)	0 (0)	0.644	-	-
	No	21 (95.5)	4 (100)			
Surgery Type	Elective	15 (68.2)	4 (100)	0.187	-	-
	Emergency	7 (31.8)	0 (0)			
Tumor Site	Right Colon	12 (54.5)	3 (75)	0.458	2.5	0.224 – 27.940
	Left Colon	10 (45.5)	1 (25)			
Tumor Size	≤5cm	10 (45.5)	1 (25)	0.446	-	-
	>5cm	12 (54.5)	3 (75)			
Tumor Appearance	Ulcero-infiltrative	18 (81.8)	4 (100)	0.651	1	0
	Polypoidal	1 (4.5)	0 (0)			
	Solid	3 (13.6)	0 (0)			
Tumor Type	Adenocarcinoma	17 (77.3)	3 (75)	0.936	0.918	0.113 – 7.469
	Mucinous	4 (18.2)	1 (25)			
	Scirrhous	1 (4.5)	0 (0)			
Differentiation	Well	15 (68.2)	2 (50)	0.249	0.267	0.028 – 2.527
	Moderate	4 (18.2)	2 (50)			
	Poor	3 (13.6)	0 (0)			
LN Stage	N0	9 (40.9)	2 (50)	0.926	1.058	0.323 – 3.463
	N1	2 (22.7)	0 (0)			
	N2	8 (36.4)	2 (50)			
Stage Group	I	1 (4.5)	0 (0)	1	-	-
	II	6 (27.3)	2 (50)			
	III	7 (31.8)	1 (25)			
	IV	8 (36.4)	1 (25)			
LVI	Evident	13 (59.1)	2 (50)	0.736	1.444	0.171 – 12.232
	Not evident	9 (40.9)	2 (50)			
PNI	Evident	0 (0)	1 (25)	0.017*	-	-
	Not Evident	22 (100)	3 (75)			

*p<0.05 to be considered significant; LN, Lymph node; LVI, Lymphovascular invasion; PNI, Perineural invasion; MSI, Microsatellite Instability; MSS, Microsatellite stable; OR, Odds Ratio; CI, Confidence interval

**Table 3:** Stage-Wise Distribution of Patients and Their Outcomes

Stage	Number of patients (n)	MSI	MSS	Disease Recurrence	Synchronous disease	Alive	Dead	Lost to Follow-up
I	1 (3.8%)	0	1	1	-	-	1#	
II	8 (30.7%)	2 (MSI-H)	6	2	-	4	2	2 (2 MSI-H after 27 months)
III	8 (30.7%)	1 (MSI-H)	7	3	-	3 (1 MSI-H)	5 *	-
IV	9 (34.6%)	1 (MSI-L)	8	-	9	-	6 (1 MSI-L)	3 (within 6 months)

# early recurrence; * including 1 perioperative death; MSS, Microsatellite stable; MSI, Microsatellite Instability; MSI-H, Microsatellite instability – High; MSI-L, Microsatellite instability – Low

**Figure 3:**
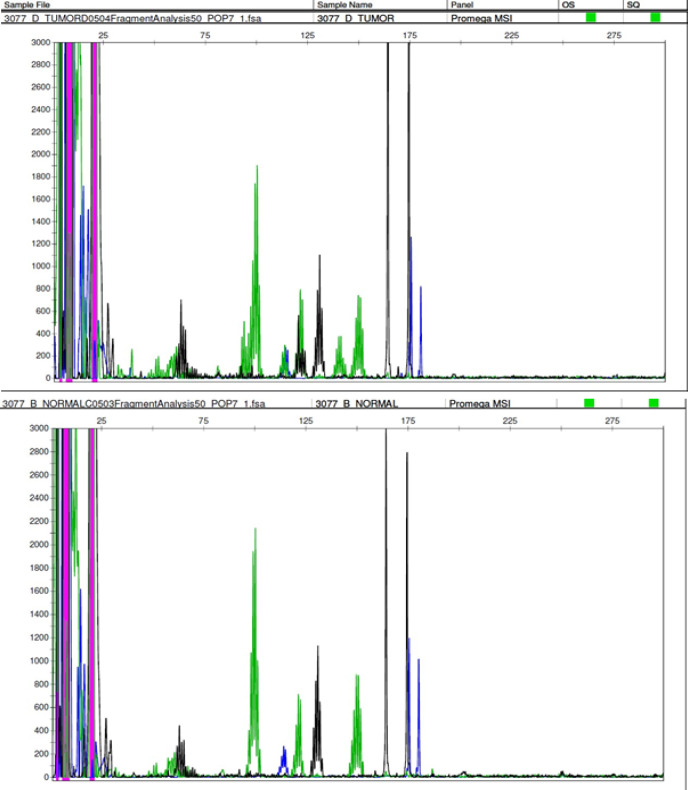
DNA Promega Panel Showing MSI-H Status

**Figure 4:**
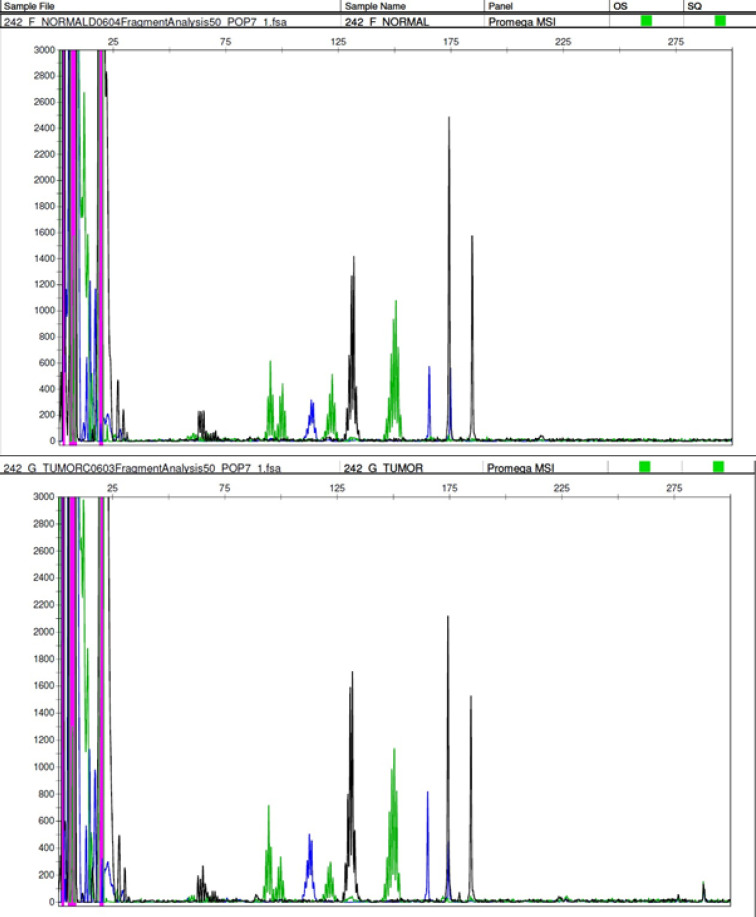
DNA Promega Panel Showing MSI Negative (MSS) Status

## Author Contribution Statement

1. Study conception and design – Dr. Smita Chauhan, Dr Pradyumn Singh, Dr. Nuzhat Hussain; 2. Data collection, analysis, manuscript drafting and critical appraisal – Dr. Smita Chauhan, Dr. Suneed Kumar, Dr Pradyumn Singh, Dr. Nuzhat Hussain, Dr. Shakeel Masood. 
